# Exercise-induced increase in muscle insulin sensitivity in men is amplified when assessed using a meal test

**DOI:** 10.1007/s00125-024-06148-x

**Published:** 2024-04-25

**Authors:** Christian T. Voldstedlund, Kim A. Sjøberg, Farina L. Schlabs, Casper M. Sigvardsen, Nicoline R. Andersen, Jens J. Holst, Bolette Hartmann, Jørgen F. P. Wojtaszewski, Bente Kiens, Glenn K. McConell, Erik A. Richter

**Affiliations:** 1 August Krogh Section for Molecular Physiology, Department of Nutrition, Exercise and Sports, Faculty of Science, University of Copenhagen, Copenhagen, Denmark; 2 Novo Nordisk Foundation Center for Basic Metabolic Research, Faculty of Health and Medical Sciences, University of Copenhagen, Copenhagen, Denmark; 3 Department of Biomedical Sciences, University of Copenhagen, Copenhagen, Denmark; 4 Institute for Health and Sport, Victoria University, Melbourne, VIC Australia

**Keywords:** Exercise, Food intake, Insulin sensitivity, Interstitial glucose, Meals, Microdialysis

## Abstract

**Aims/hypothesis:**

Exercise has a profound effect on insulin sensitivity in skeletal muscle. The euglycaemic–hyperinsulinaemic clamp (EHC) is the gold standard for assessment of insulin sensitivity but it does not reflect the hyperglycaemia that occurs after eating a meal. In previous EHC investigations, it has been shown that the interstitial glucose concentration in muscle is decreased to a larger extent in previously exercised muscle than in rested muscle. This suggests that previously exercised muscle may increase its glucose uptake more than rested muscle if glucose supply is increased by hyperglycaemia. Therefore, we hypothesised that the exercise-induced increase in muscle insulin sensitivity would appear greater after eating a meal than previously observed with the EHC.

**Methods:**

Ten recreationally active men performed dynamic one-legged knee extensor exercise for 1 h. Following this, both femoral veins and one femoral artery were cannulated. Subsequently, 4 h after exercise, a solid meal followed by two liquid meals were ingested over 1 h and glucose uptake in the two legs was measured for 3 h. Muscle biopsies from both legs were obtained before the meal test and 90 min after the meal test was initiated. Data obtained in previous studies using the EHC (*n*=106 participants from 13 EHC studies) were used for comparison with the meal-test data obtained in this study.

**Results:**

Plasma glucose and insulin peaked 45 min after initiation of the meal test. Following the meal test, leg glucose uptake and glucose clearance increased twice as much in the exercised leg than in the rested leg; this difference is twice as big as that observed in previous investigations using EHCs. Glucose uptake in the rested leg plateaued after 15 min, alongside elevated muscle glucose 6-phosphate levels, suggestive of compromised muscle glucose metabolism. In contrast, glucose uptake in the exercised leg plateaued 45 min after initiation of the meal test and there were no signs of compromised glucose metabolism. Phosphorylation of the TBC1 domain family member 4 (TBC1D4; p-TBC1D4^Ser704^) and glycogen synthase activity were greater in the exercised leg compared with the rested leg. Muscle interstitial glucose concentration increased with ingestion of meals, although it was 16% lower in the exercised leg than in the rested leg.

**Conclusions/interpretation:**

Hyperglycaemia after meal ingestion results in larger differences in muscle glucose uptake between rested and exercised muscle than previously observed during EHCs. These findings indicate that the ability of exercise to increase insulin-stimulated muscle glucose uptake is even greater when evaluated with a meal test than has previously been shown with EHCs.

**Graphical Abstract:**

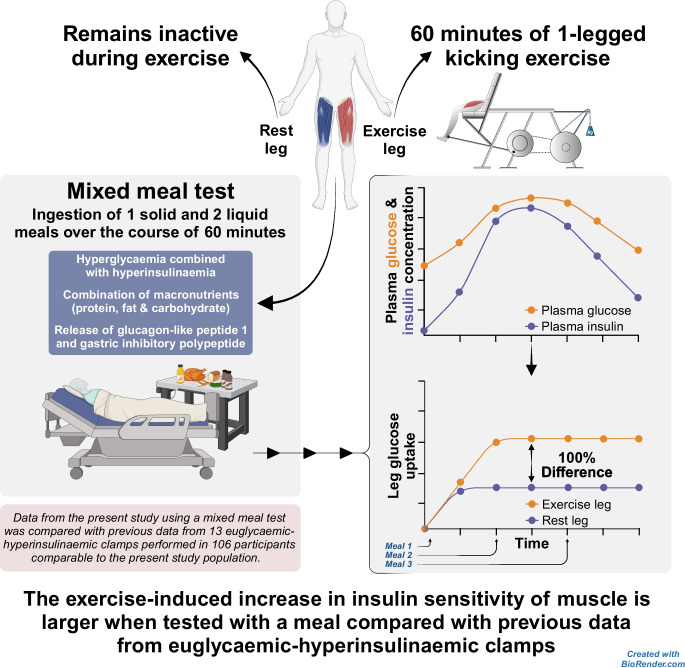

**Supplementary Information:**

The online version contains peer-reviewed but unedited supplementary material available at 10.1007/s00125-024-06148-x.


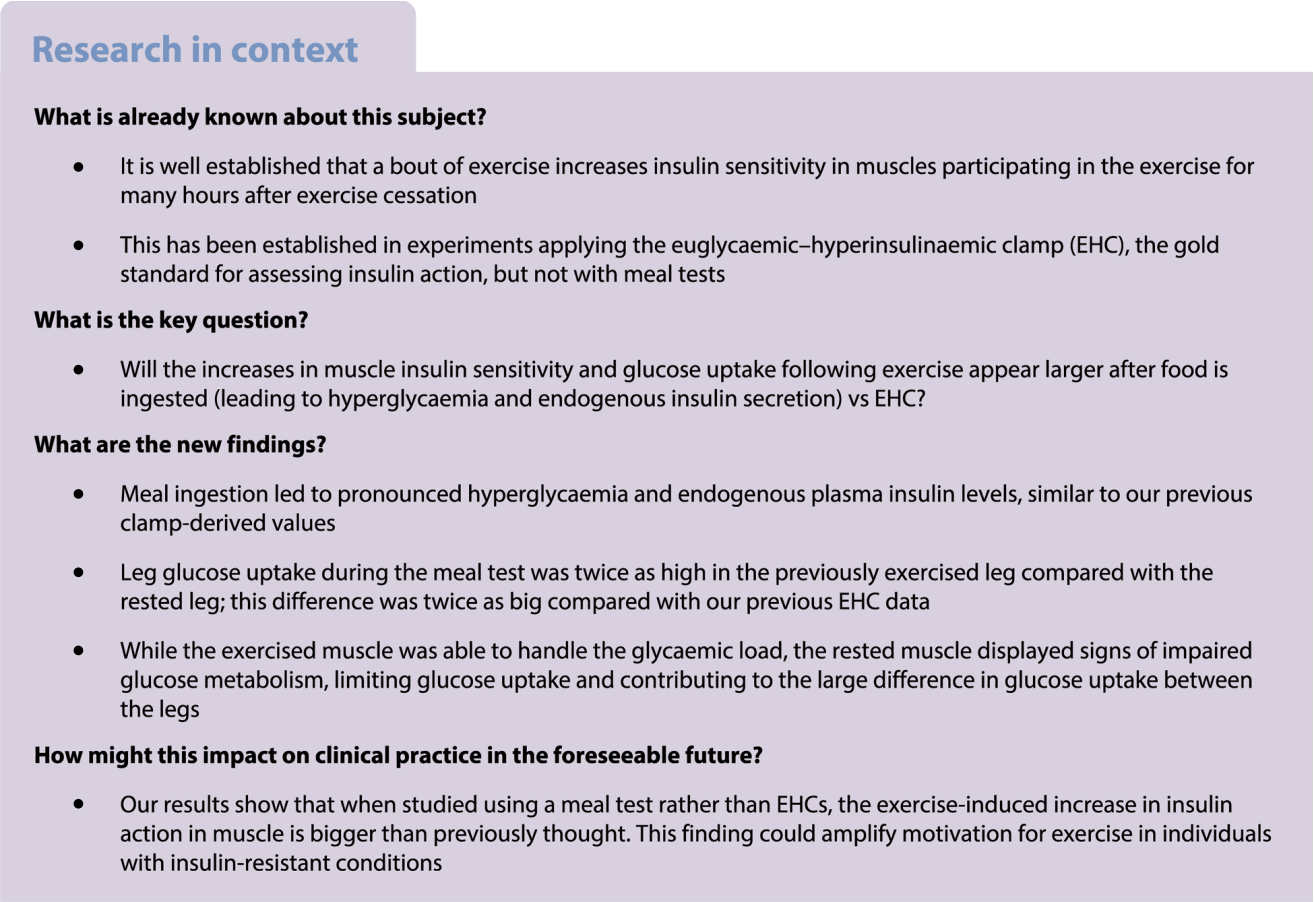


## Introduction

Exercise improves metabolic control in part by increasing muscle glucose uptake during muscle contractions by insulin independent mechanisms and by increasing skeletal muscle insulin sensitivity following exercise [1–5]. Indeed, skeletal muscle remains more sensitive to insulin after a single bout of exercise in both rodents [1, 6] and humans [3, 7, 8]. The complexity of the exercise-induced increase in insulin sensitivity is highlighted in individuals with type 2 diabetes, for whom muscle tissue does not respond normally to insulin, yet their skeletal muscle glucose uptake is normal during exercise [9, 10] and they appear to exhibit normal increases in insulin sensitivity after exercise [11].

Most of our knowledge concerning insulin sensitivity in healthy and patient populations with and without exercise has been obtained using the gold standard measure, the euglycaemic–hyperinsulinaemic clamp (EHC) [12]. Although the EHC is a valuable tool to assess insulin sensitivity, it should be emphasised that the methodology is non-physiological in nature, due to euglycaemia and a high constant level of insulin being maintained due to infusion [13]. This means that the pancreatic beta cells and the gut are bypassed, which contrasts with the physiological scenario of ingesting a meal. In addition, the metabolic effects of increases in glucose and amino acids, activation of mouth macronutrient receptors [14], the gut microbiota [15] and incretin hormones, such as glucagon-like peptide 1 (GLP-1) [16] and gastric inhibitory polypeptide (GIP) [17], which are released with ingestion of a meal, are all bypassed by an EHC due to the systemic infusion approach. It is likely that many of these normal physiological signals have effects on glucose uptake, insulin signalling and blood flow that are not seen with the EHC.

Even though total leg blood flow (LBF) and muscle microvascular perfusion increase during an EHC and more so in a previously exercised leg [5, 18], it appears that the increase in glucose transport and glucose metabolism surpasses glucose delivery to the leg given that we [19] and others [20] have observed that skeletal muscle interstitial glucose concentration decreases during the EHC, especially in a previously exercised muscle [19]. It is possible that the reductions in skeletal muscle interstitial glucose concentration are artefacts of the EHC because the high insulin concentration strongly stimulates glucose uptake, but glucose delivery is limited because the plasma glucose concentration is kept at euglycaemia and therefore does not match the high insulin concentration. Further, the lower interstitial glucose concentration in the exercised compared with the rested leg suggests that if glucose delivery is increased, the potential for increasing glucose uptake may be larger in the previously exercised leg. It is, therefore, important to determine whether meal ingestion is associated with increases in skeletal muscle interstitial glucose and whether such increases lead to greater enhancement of glucose uptake in exercised than in rested muscle.

Therefore, the aim of this study was to determine the ability of insulin to increase leg glucose uptake following exercise during hyperglycaemia resulting from ingestion of meals. We hypothesised that meal ingestion would raise skeletal muscle interstitial glucose due to a concomitant increase in glucose delivery together with insulin and that the exercise-induced increase in muscle insulin sensitivity evaluated by leg glucose uptake would be greater when measured with a meal test than previously observed with the EHC.

## Methods

### Participants and ethics

Ten recreationally active and healthy white men participated in the study (Table 1), which was approved by the Research Ethics Committee of Copenhagen (H-18006850) and complied with the Declaration of Helsinki. Race and sex were self-reported. Exclusion criteria were BMI>25 kg/m^2^, self-reported activity level >10 h per week, use of medication or immediate family history of diabetes.

**Table 1:** Participant characteristics

Characteristic	Mean±SEM
Age (years)	26.7±1.3
Body weight (kg)	74.9±2.4
BMI (kg/m^2^)	22.6±0.6
Body fat (%)	18.9±1.3
Visceral fat mass (g)	287±56
Whole body lean mass (kg)	57.8±2.0
Lean mass (kg)	
Rest leg	9.8±0.4
Exercise leg	9.9±0.4
$\dot{V}$O_2 peak_ (ml kg^−1^ min^−1^)	44.2±1.6
Peak knee extensor workload (W)	42.4±3.3
Exercise workload (W)	33.8±2.6

Data presented for *n*=10 participants

### Screening and preliminary tests

Participants were screened by an incremental $\dot{V}{\text{O}}_{\text{2peak}}$ test (Vyntus CPX; Vyaire Medical, USA) on a cycle ergometer (Monark E839; Monark, Sweden) after which they were familiarised to one-legged knee extensor exercise. On a separate day, under fasting conditions, body composition was determined by dual-energy x-ray absorptiometry (DPX-IQ Lunar; Lunar, USA) and a peak-workload test was performed on the one-legged knee extensor apparatus. The peak-workload test was performed at least 7 days prior to the experimental trial and half of the participants were randomly assigned to use their non-dominant leg.

Participants were instructed to eat their habitual diet and refrain from exercise training 2 days prior to the experimental day. Participants arrived by car or public transportation on the experimental day.

### Experimental day

At 06:00 hours, prior to arrival, participants ingested a standardised (18 kJ/kg) breakfast of oats, skimmed milk and sugar to avoid large excursions in plasma fatty acid levels due to prolonged fasting.

Participants arrived at 07:30 hours and commenced 60 min of continuous one-legged knee extensor exercise (3×20 min blocks composed of 15 min at 80% and 5 min at 100% peak workload). The contralateral leg remained inactive. Subsequently, participants rested for 4 h in the supine position for the LBF and glucose uptake to reach comparable levels in both legs, similar to pre-exercise levels. Meanwhile, catheters were inserted into one femoral artery and both femoral veins below the inguinal ligament (Pediatric Jugular Catheterization set; Arrow International, USA) under local anaesthesia (Xylocaine 1% [wt/vol.]; AstraZeneca, Denmark) for blood sampling.

### Microdialysis

**Fig. 1:**
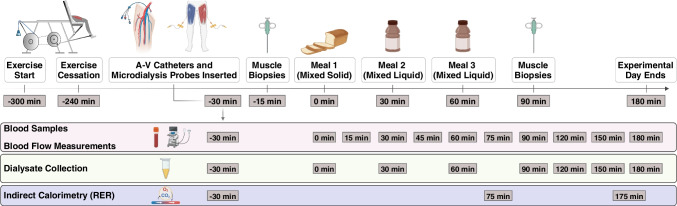
Schematic overview of the experimental protocol. Created with BioRender.com. RER, respiratory exchange ratio

Microdialysis for skeletal muscle interstitial glucose measurements was undertaken as we have described in detail previously [19]. In brief, approximately 1.5 h after completion of exercise, two microdialysis catheters (M Dialysis, Sweden) were inserted into the vastus lateralis muscle of both legs under local anaesthesia of the skin and the fascia, approximately 20 cm apart. The perfusate was composed of 10 ml of saline (154 mmol/l NaCl) with 1 mmol/l d-glucose and 1.1 μl of sterile labelled glucose tracer (d-[6-^3^H(N)]glucose) containing 0.041 MBq activity. The perfusion was initiated with a bolus infusion of 10 μl/min for 10 min followed by a constant infusion of 1 μl/min using micro infusion pumps (Pump 11 Plus; Harvard Apparatus, USA) for 90 min to ensure equilibration between perfusate and interstitial fluid [21]. Thereafter, dialysate was collected every 30 min throughout the experiment (Fig. 1) and analysed for glucose concentration on a glucometer (Blood Glucose Meter; Bayer, Switzerland), and disintegrations per min (DPM) were counted in a liquid scintillation analyser (Tri-Carb 2910 TR; Perkin Elmer, USA). The difference in DPM between the dialysate and perfusate was used to calculate the recovery rate for the glucose concentration between the dialysate and the interstitial fluid [19, 22] and to further calculate the interstitial glucose concentration [23]. When comparing the interstitial glucose concentration with the concentration in plasma, the arterial concentration of glucose was corrected to plasma water by dividing by 0.94 [20, 24, 25]. The results from the two probes in each leg were pooled into a single mean value for each time point. The mean recovery rate of the microdialysis probes was 0.86±0.03 for both legs. Since dialysate was collected over the course of 30 min, the samples reflect the average concentration of the time interval. For this reason, time points are shown as the midpoint of the collection interval (i.e. dialysate samples collected at 30 min reflect the concentration between time point 0 min and 30 min and is therefore shown as 15 min).

### Meals

Meal ingestion was initiated 4 h after exercise cessation (time point ‘0 min’) by ingestion of a mixed solid meal (30 kJ per kg body weight). Subsequently, participants ingested a mixed liquid meal (20 kJ per kg body weight) (Nutridrink; Nutrica, Denmark) 30 min and 60 min after ingestion of the solid meal to achieve hyperglycaemia and endogenous hyperinsulinaemia. All meals were ingested within 5 min and were composed of ~50% of total energy intake (E%) carbohydrates, ~35 E% fat and ~15 E% protein, to match the habitual energy composition of the Danish population [26, 27] and to provide a substantial energy load. The larger first meal and the subsequent two smaller meals were chosen in an effort to reach a fairly stable plasma level of glucose and insulin in the 30–90 min interval after the first meal to approximate steady-state conditions.

### Skeletal muscle biopsies

Muscle biopsies were obtained from the vastus lateralis muscle of both legs under local anaesthesia of the skin and the fascia using the Bergstrom needle technique with suction, prior to initiation of the meal test and 90 min after ingestion of the first meal, from separate incisions spaced 5–6 cm apart.

### Femoral artery blood flow

LBF in both legs was measured immediately before blood sampling using a high-frequency 12–3 MHz linear array transducer in Power Doppler mode interfaced to an Affiniti70 ultrasound machine (both Phillips Ultrasound, USA), as described previously [28].

### Blood sampling, leg glucose uptake and glucose clearance

Plasma glucose and lactate concentrations were measured by use of an ABL 800 FLEX (Radiometer Medical, Denmark). Lean leg mass (LLM) glucose uptake from 0 min to 180 min was calculated by dividing the product of the glucose arterial-venous (A-V) difference and the LBF with the lean mass of the leg. Glucose clearance was determined by dividing the leg glucose uptake by the arterial plasma glucose at the same time point.

### Plasma analysis

Plasma insulin was measured using a sandwich type ELISA (Alpco, USA). Plasma fatty acids (NEFA C Kit; Wako Chemicals, Germany) were measured using colorimetric methods on an autoanalyser (Pentra C400; Horiba, Japan). Total GLP-1 was determined using an in-house radioimmunoassay targeting the amidated C terminus of GLP-1 [29], while total GIP was measured using a commercially available sandwich ELISA kit (catalogue no. 10-1258-01; Mercodia, Sweden).

### Muscle processing—homogenisation

First, 25 mg of muscle was freeze dried, dissected and homogenised (Tissuelyser II; Qiagen, Germany) in fresh ice-cold buffer as previously described [30]. After homogenisation, samples were turned end-over-end at 4°C for 1 h followed by centrifugation at 4°C at 16,000 *g* for 20 min (Universal 320R; Hettich, Denmark). Protein content was determined in triplicate using a bicinchoninic assay method with BSA as standard and absorbance measurements by spectrophotometry (Multiscan FC; Thermo Fisher Scientific, USA). Upon determination of protein content, lysates were mixed with 6× Laemmli buffer.

### SDS-PAGE and western blotting

Proteins were separated by SDS-PAGE and semi-dry transferred (Trans-Blot Turbo; Bio-Rad Laboratories, USA) onto PVDF membranes (Immobilon-P; Merck Millipore, USA). Membranes were blocked (3% [wt/vol.] BSA or 2% [wt/vol.] skimmed milk) in TBS with Tween 20 (TBST) buffer before incubation overnight at 4°C in primary antibody diluted in 3% [wt/vol.] BSA or 2% [wt/vol.] skimmed milk (electronic supplementary material [ESM] Table 1). Membranes were washed in TBST before incubation with the relevant secondary antibody for 45 min and were visualised using enhanced chemiluminescence (Immobilon-Forte; Merck Millipore) on a ChemiDoc XRS+ (Bio-Rad Laboratories). Band intensity was determined with Image Lab version 6.1 (Bio-Rad Laboratories). Validation of loading and protein transfer was performed by use of the Pierce Reversible Protein Stain Kit (Thermo Fisher Scientific).

### Muscle glycogen, glycogen synthase activity, muscle glucose and glucose 6-phosphate

Muscle glucose and glucose 6-phosphate concentrations were determined (Fluoroskan; Thermo Fisher Scientific) by use of standard enzymatic methods while muscle glycogen concentration was determined as glycosyl units after acid hydrolysis by a fluorometric method [31]. Glycogen synthase activity was measured in muscle homogenates using a Unifilter 350 microtitre plate assay (Whatman, Denmark), essentially as described by Thomas et al [32] but modified for microtitre plate assay [33]. Glycogen synthase activity was measured in presence of 0.02 mmol/l (I-form) and 0.17 mmol/l (fractional velocity % [FV%]) glucose 6-phosphate. The measures at both concentrations were multiplied by 100 and divided by total glycogen synthase activity at a glucose 6-phosphate concentration of 8 mmol/l.

### Statistical analysis

Data are presented as mean±SEM. Statistical significance was determined by use of repeated measures one- or two-factor analysis of variance. Correction for multiple comparisons was performed by use of the Holm–Šídák method in case of significant main effects and interactions. A Geisser–Greenhouse correction was used in case of sphericity violations. The α level was set to *p*<0.05. Statistical analyses were performed using GraphPad Prism version 10.0.2 for Windows (GraphPad Software, USA).

## Results

### Plasma glucose, femoral artery blood flow, A-V difference, leg glucose uptake and glucose clearance

**Fig. 2:**
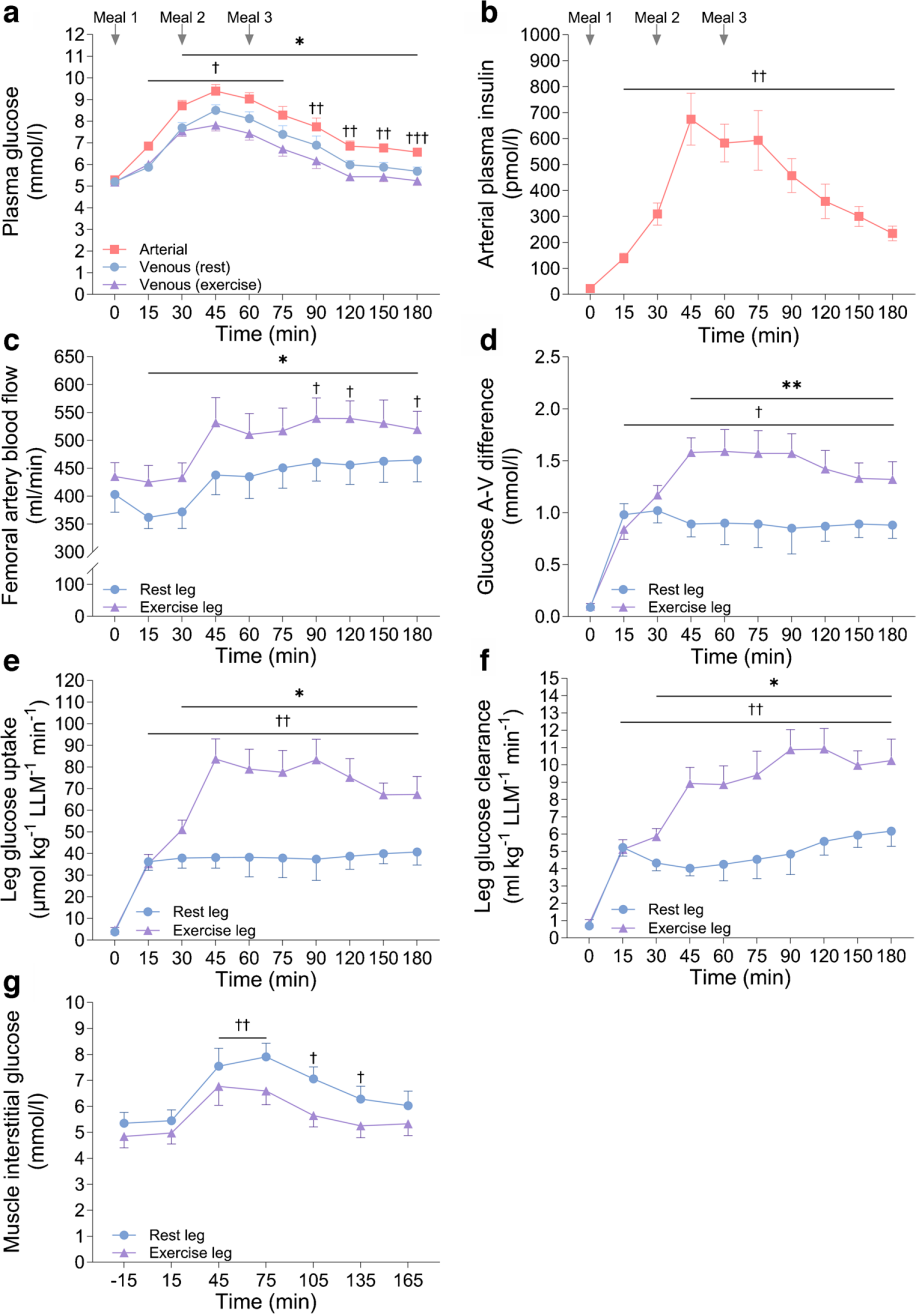
(**a**) Plasma glucose concentration in the femoral artery and veins of both legs. (**b**) Arterial plasma insulin concentration. (**c**) Femoral artery blood flow. (**d**) A-V glucose difference. (**e**) Leg glucose uptake. (**f**) Leg glucose clearance measured during a mixed-meal test (0–180 min). (**g**) Interstitial glucose concentration in skeletal muscle measured during a mixed-meal test (−15 to 165 min). Data are presented as mean±SEM; *n*=10. *p<0.05, **p<0.01 for all data points under horizontal line vs corresponding time point(s) for all other condition(s) on the same graph (note, in **d**, *p*=0.064 at 30 min for rest leg vs exercise leg); ^†^*p*<0.05, ^††^*p*<0.01, ^†††^*p*<0.001 for all data points under horizontal line/at the specific time point vs within-condition baseline (0 min); analysed by one- (**b**) or two-factor (**a**, **c**–**g**) analysis of variance and a Holm–Šídák post hoc test

Arterial plasma glucose was 5.3±0.1 mmol/l 4 h after one-legged knee extensor exercise, and there was no significant difference in femoral artery LBF, A-V glucose difference or leg glucose uptake between the legs (Fig. 2a,c–e). Leg glucose uptake increased (*p*<0.01) similarly, by ~7-fold, in the rested and exercised legs 15 min after ingestion of the first meal (36±4 vs 35±4 µmol kg^−1^ LLM^−1^ min^−1^, respectively; Fig. 2e). This was reflected in a rise in arterial plasma glucose that exceeded the increase in venous glucose (Fig. 2a) such that there was a large increase in A-V glucose difference in both legs. At the same time, LBF in the exercised leg was 17% higher than the rested leg (*p*<0.05) (Fig. 2c).

Plasma glucose reached a peak of 9.4±0.3 mmol/l 45 min after initiation of meal ingestion (Fig. 2a). Unlike the resting leg, where no further increase in leg glucose uptake was observed after 15 min, glucose uptake in the exercised leg at 45 min after initiation of meal ingestion increased to a level that was 119% higher than the rested leg (*p*<0.01) (Fig. 2e). This was mainly due to a much larger glucose extraction in the exercised leg, with A-V glucose difference doubling from 15 min to 45 min in that leg while it did not change in the rested leg (Fig. 2d). The greater leg glucose uptake in the exercised leg at 45 min was also partly due to a 22% greater LBF in the exercised than the rested leg (Fig. 2c). Although arterial plasma glucose concentration began to decline 60 min after initiation of meal ingestion, leg glucose uptake remained essentially unchanged due to a steady LBF combined with a parallel decrease in arterial and venous plasma glucose concentration (Fig. 2a) resulting in a relatively constant A-V glucose difference (Fig. 2d). Glucose clearance was greater in the exercised leg than the rested leg from 15 min onwards (Fig. 2f).

The present data were compared with findings from our previously published study (ESM Fig. 1a and [5]) averaging data from 13 previous EHC investigations (*n*=106 participants) performed after one-legged exercise. The EHC-study participants appeared to have similar characteristics to the participants in the present study (see ESM Table 2 and Table 1). It is apparent that the time course of the increase in leg glucose uptake is different compared with the EHC and that the difference in leg glucose uptake between the previously exercised and the rested leg is about twice as large after ingestion of a meal than during the EHC.

### Plasma insulin

Arterial plasma insulin increased ~7-fold within 15 min of the first meal and then increased greatly and close to linearly to about 30-fold 45 min after meal ingestion (Fig. 2b). Despite a third meal 60 min after ingestion of the first meal, plasma insulin decreased linearly from 75 min (Fig. 2b). From 45 min to 75 min, the plasma insulin concentration appeared to be similar to the levels we observed during our previous EHC studies after exercise (~600 pmol/l) [5].

### Interstitial glucose

Although numerically lower in the exercised leg, vastus lateralis interstitial glucose was not statistically different between the legs before meal ingestion at 4 h after one-legged exercise (Fig. 2g). There was no change in interstitial glucose concentration over the first 15 min after meal ingestion. It then increased with meal ingestion in both legs and, despite a higher glucose delivery in the previously exercised leg (higher blood flow), the mean interstitial glucose AUC was 16% lower (*p*<0.05) in the previously exercised leg (rested leg vs exercised leg: 39.9±2.8 vs 34.4±2.8 arbitrary units [AU]). This difference indicates the increased ability of exercised compared with rested muscle to take up glucose from the interstitial space. This increase in interstitial glucose concentrations appeared to be quite different compared with the decrease in interstitial glucose concentrations obtained in our previous EHC study (ESM Fig. 1b and [19]).

### Plasma GLP-1 and GIP

**Fig. 3:**
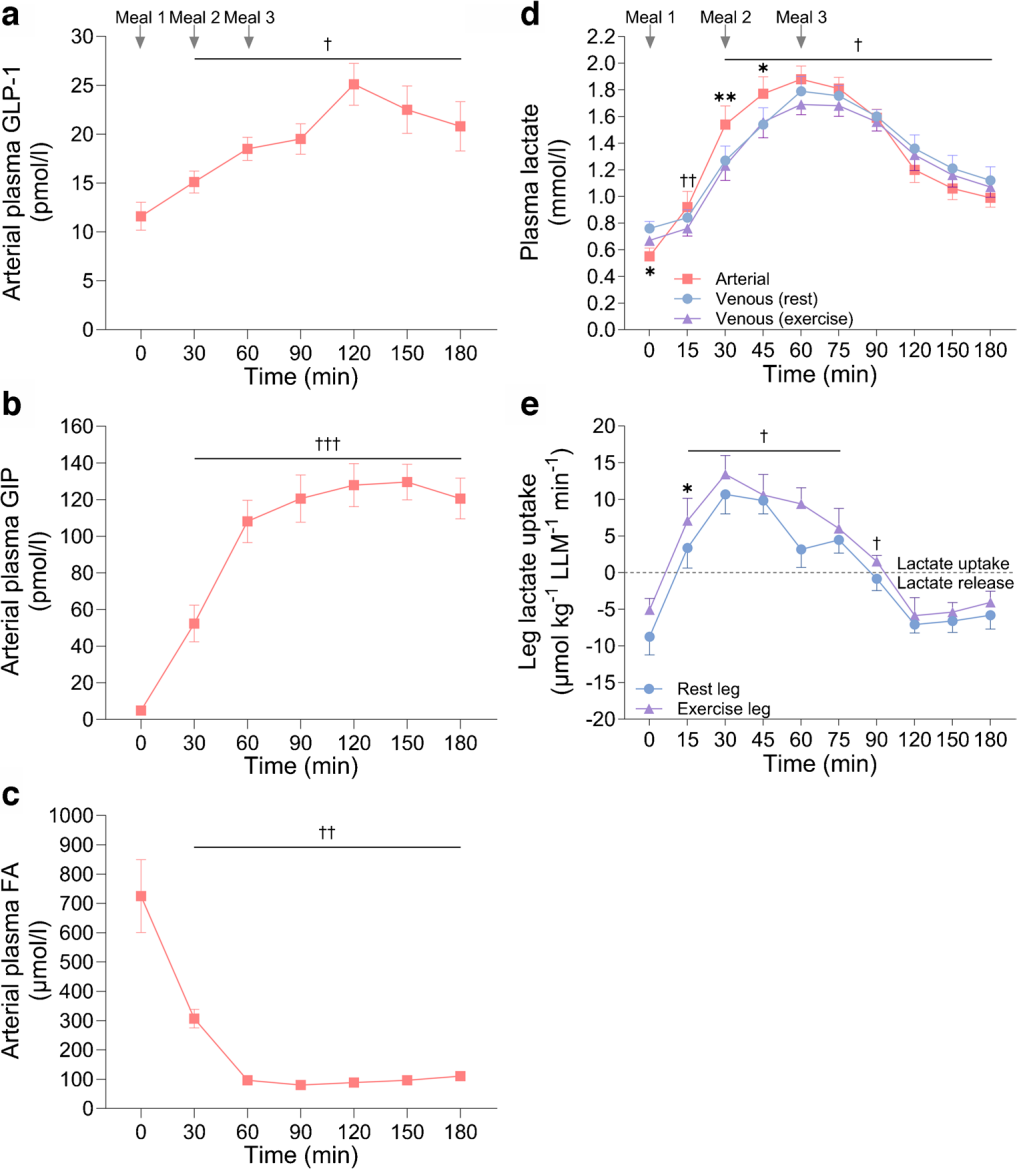
(**a**) Arterial plasma GLP-1 concentration. (**b**) Arterial plasma GIP concentration. (**c**) Arterial plasma fatty acid (FA) concentration. (**d**) Plasma lactate concentration in the femoral artery and veins of both legs. (**e**) Leg lactate uptake measured during a mixed-meal test (0–180 min). Data above the dotted horizontal line indicate lactate uptake; data below the dotted horizontal line indicate lactate release. Data are presented as mean±SEM; *n*=10. *p<0.05, **p<0.01 vs corresponding time point(s) for all other condition(s) on the same graph; ^†^*p*<0.05, ^††^*p*<0.01, ^†††^*p*<0.001 for all data points under solid horizontal line/at the specific time point vs within-condition baseline (0 min); analysed by one- (**a**–**c**) or two-factor (**d**, **e**) analysis of variance and a Holm–Šídák post hoc test

Meal ingestion resulted in a close to linear increase in arterial plasma GLP-1 with an approximate doubling (*p*<0.01) observed at 120 min, after which it decreased (Fig. 3a). In contrast, arterial GIP increased (*p*<0.001) around 20-fold 60 min after meal ingestion, after which it plateaued (Fig. 3b).

### Plasma fatty acids

Arterial plasma fatty acid levels (mean ±SEM) were 725±124 µmol/l 4 h after exercise, and decreased markedly (*p*<0.01) at 30 min and then further at 60 min post meal, after which it remained relatively stable for the remainder of the trial (Fig. 3c).

### Plasma lactate

Intriguingly, meal ingestion induced a marked increase in plasma lactate, with mean arterial concentration increasing from 0.6±0.1 mmol/l to a peak of 1.9±0.1 mmol/l (*p*<0.001) 60 min after initiation of meal ingestion (Fig. 3d). Moreover, a net leg lactate uptake was observed in both legs from 15 min until 90 min with a 36% higher mean uptake in the previously exercised leg (AUC *p*<0.05; Fig. 3e), indicating that the increase in plasma lactate was due to release from non-leg tissues.

### Muscle glycogen, glucose, glucose 6-phosphate and glycogen synthase

**Fig. 4:**
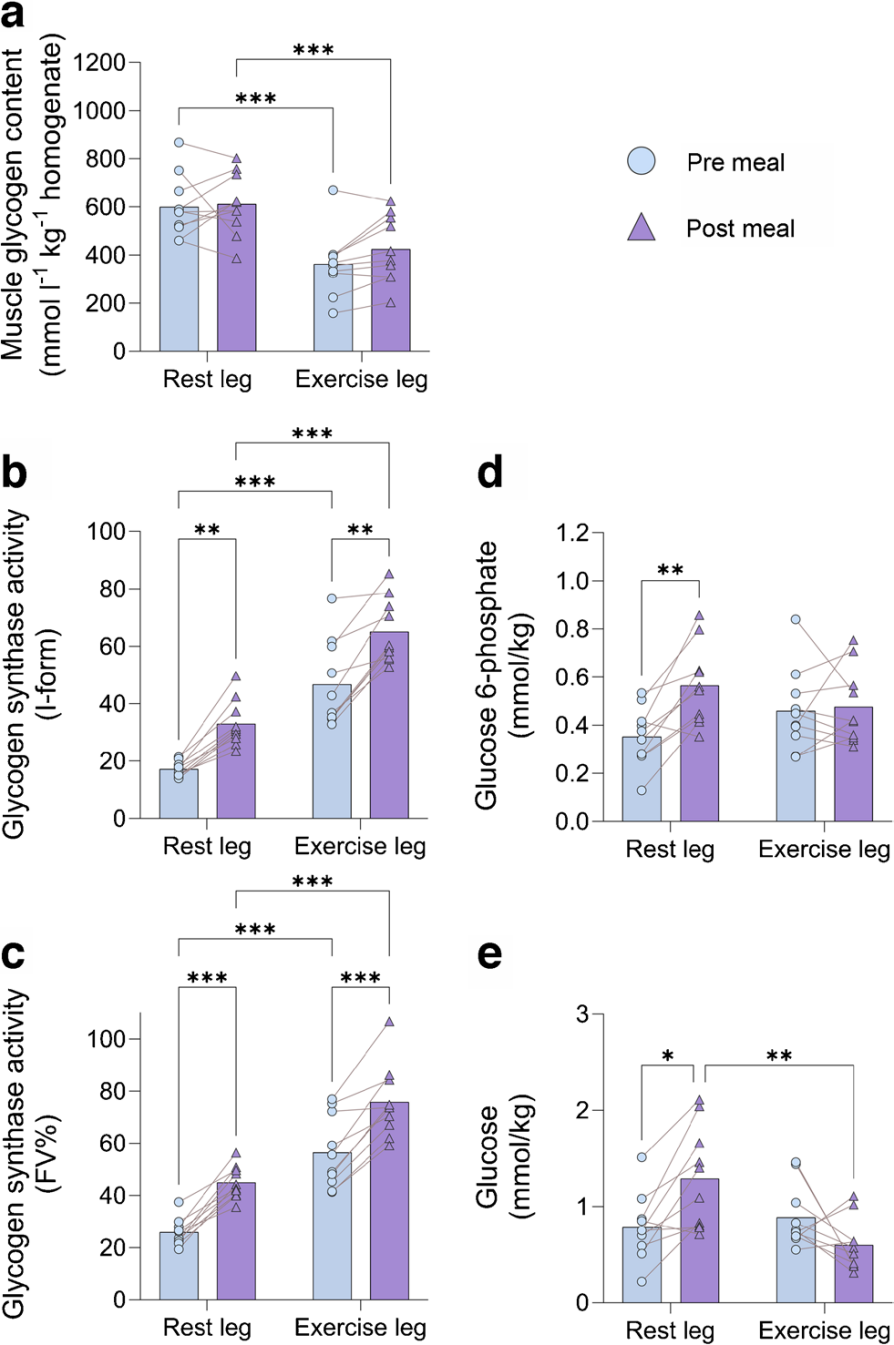
(**a**) Muscle glycogen content expressed as mmol l^-1^ kg^-1^ homogenate protein. (**b**) Glycogen synthase activity (I-form; activity measured in presence of 0.02 mmol/l glucose 6-phosphate, multiplied by 100 and divided by total glycogen synthase activity at a glucose 6-phosphate concentration of 8 mmol/l). (**c**) Glycogen synthase activity (fractional velocity [FV%]; activity measured in presence of 0.17 mmol/l glucose 6-phosphate, multiplied by 100 and divided by total glycogen synthase activity at a glucose 6-phosphate concentration of 8 mmol/l). (**d**) Muscle glucose 6-phosphate content. (**e**) Free glucose content in muscle. Data are presented as mean±SEM with individual data points shown; *n*=10. *p<0.05, **p<0.01, ***p<0.001, analysed by two-factor analysis of variance and a Holm–Šídák post hoc test

Compared with the rested leg, muscle glycogen content was lower and muscle glycogen synthase activity higher in the exercised leg 4 h after the one-legged exercise (Fig. 4a–c). Meal ingestion had no significant effect on muscle glycogen content in either leg but increased muscle glycogen synthase activity similarly by ~18% in both legs, with muscle glycogen synthase activity remaining higher in the exercised leg. There were no significant differences between legs in muscle glucose or glucose 6-phosphate concentration 4 h after the one-legged exercise (Fig. 4d,e). Meal ingestion increased muscle glucose (~65%) and glucose 6-phosphate concentration (~60%) in the rested leg, while in the exercised leg, meal ingestion caused an absolute yet non-significant decrease in muscle glucose concentration by ~33% while there was no change in muscle glucose 6-phosphate concentration. As a result, a lower muscle glucose concentration was observed in the previously exercised leg compared with the rested leg (*p*<0.05) 90 min after meal ingestion. These data suggest that glucose metabolism is limiting muscle glucose uptake in the rested leg.

### Signalling for muscle glucose transport and protein synthesis

**Fig. 5:**
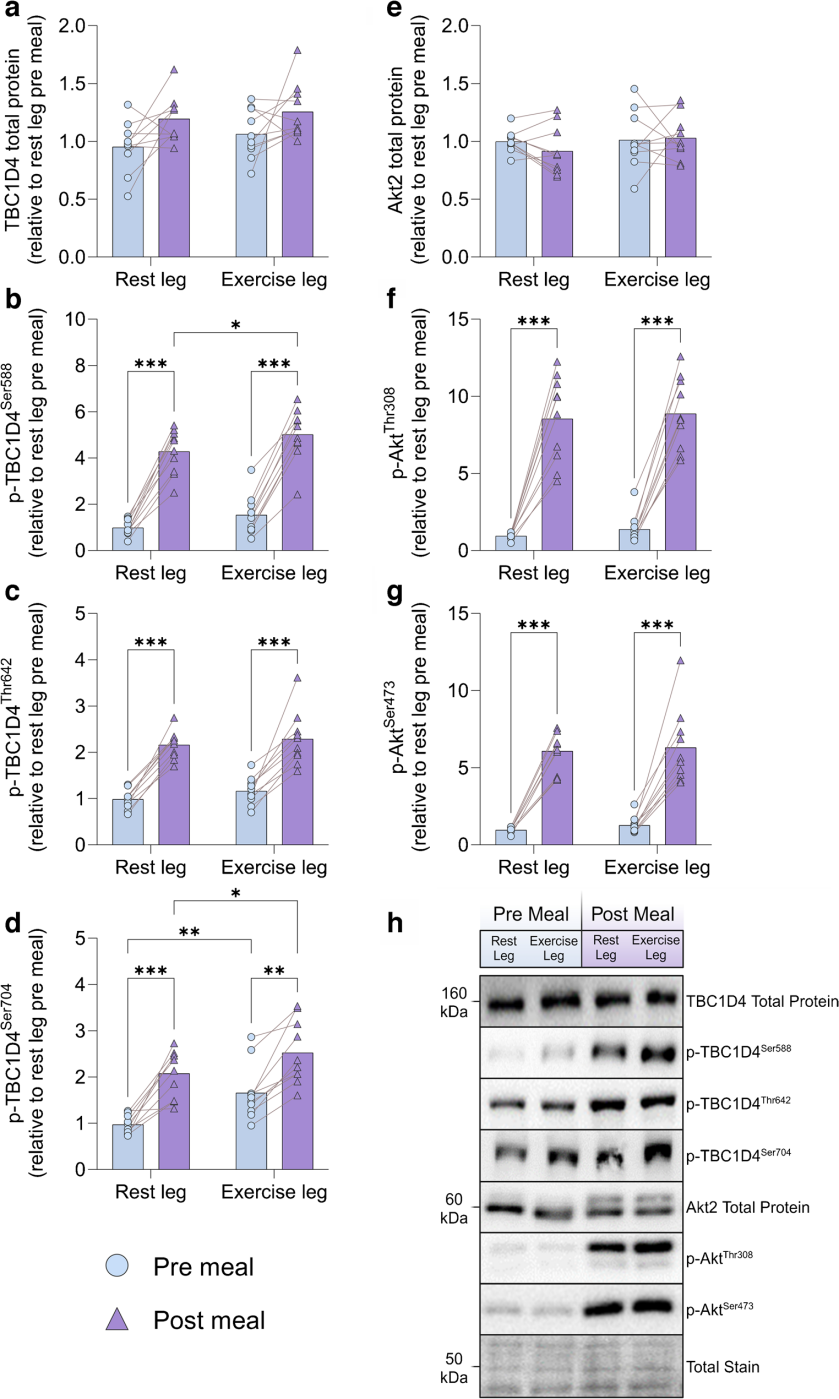
Skeletal muscle molecular signalling in the fasting state and in response to a mixed-meal test measured by western blotting is shown. (**a**) TBC1D4 total protein. (**b**) p-TBC1D4^Ser588^. (**c**) p-TBC1D4^Thr642^. (**d**) p-TBC1D4^Ser704^. (**e**) Akt2 total protein. (**f**) p-Akt2^Thr308^. (**g**) p-Akt2^Ser473^. (**h**) Representative western blots for all proteins analysed. Data are presented as means with individual data points shown; data are normalised to the mean band intensity of all basal (pre meal, rest leg) measures on the gel that the protein was loaded; *n*=10. *p<0.05, **p<0.01, ***p<0.001, analysed by two-factor analysis of variance and a Holm–Šídák post hoc test

There was no difference in the total protein content of Akt2 between the legs while a main effect of exercise (*p*<0.05) was observed for TBC1 domain family member 4 (TBC1D4) total protein content (Fig. 5a,e). Exercise had no effect on p-Akt^Thr308^, p-Akt^Ser473^ or p-TBC1D4^Thr642^, while meal ingestion increased these to a similar extent in both legs (Fig. 5c,f,g). Exercise and meal ingestion increased p-TBC1D4^Ser704^ and a potentiating effect of exercise with meal ingestion (*p*<0.05) was observed for p-TBC1D4^Ser588^ (Fig. 5b,d). Exercise has previously been shown to increase insulin sensitivity of signalling controlling protein synthesis with the EHC [34, 35]. Here there was no effect of exercise or meal ingestion on p70 ribosomal protein S6 kinase (p70S6K) or Unc-51-like kinase 1 (ULK1) total protein content (ESM Fig. 2b,d). Exercise alone had no effect on phosphorylation of the mammalian target of rapamycin complex 1 (mTORC1; p-mTORC1^Ser2448^), or on p-p70S6K^Thr389^ or p-ULK1^Ser757^ (ESM Fig. 2a,c,e). Ingestion of the meals significantly increased p-mTORC1^Ser2448^ in both legs (ESM Fig. 2a), while p-p70S6K^Thr389^ was increased to a greater extent in the exercised leg (ESM Fig. 2c) and p-ULK1^Ser757^ was only increased in the exercised leg (ESM Fig. 2e).

## Discussion

Following exercise, muscle insulin sensitivity is increased compared with non-exercised muscle. This has been demonstrated many times with the EHC at euglycaemia with approximately 600 pmol/l plasma insulin concentration combined with leg catheterisation, where leg glucose uptake is about 55% higher in the exercised compared with the rested leg ([3, 5, 18, 19] and ESM Fig. 1a). In contrast, in the present study the difference between the exercised and the rested leg glucose uptake was about 100% (Fig. 2e), i.e. twice as large a difference than previously observed during the EHC (ESM Fig. 1a). This is likely due to the hyperglycaemia observed in the present study as plasma insulin concentrations appeared to be similar to previous EHC-derived values, at least during the time interval between 45 min and 75 min after the meal ingestion [5]. The importance of hyperglycaemia vs euglycaemia is also reflected in the difference in the skeletal muscle interstitial glucose concentrations, which in both legs decreased in our previously published EHC study ([19] and ESM Fig. 1b) while they increased in the present study (Fig. 2g). This shows that the glucose concentration on the outside of the myocyte is significantly higher with meal ingestion than during an EHC.

**Fig. 6:**
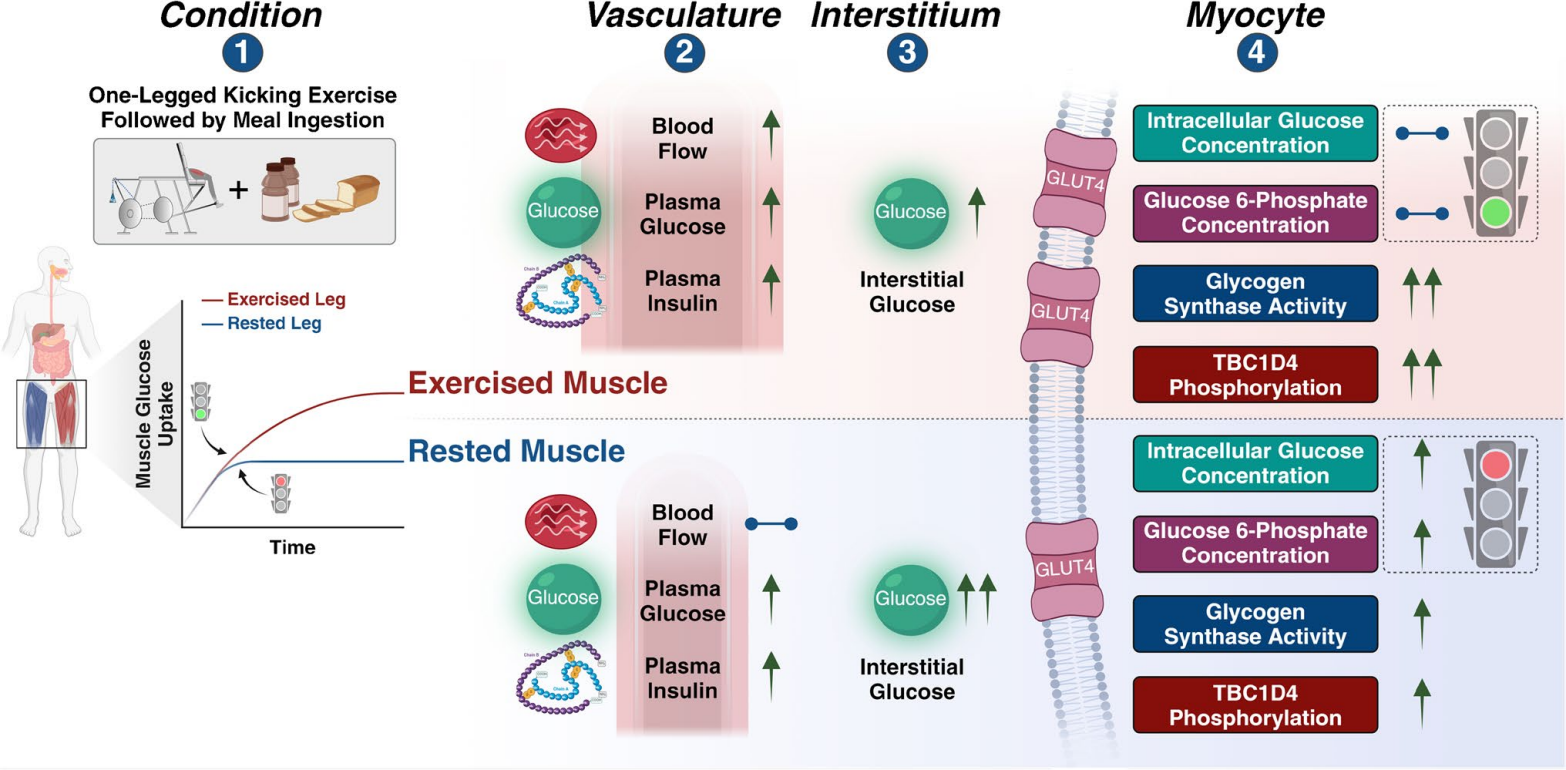
A schematic of the effect of meal ingestion on metabolism after one-legged exercise in the previously exercised leg and the rested leg. All variables shown were measured in this study except for plasma membrane/transverse tubule (T-tubules) GLUT4 translocation, which was estimated based on the insulin signalling results and our previous studies [44, 45]. Traffic lights indicate processes that result in either increased (green light) or decreased (red light) flux. Created with BioRender.com

In the present study a reasonable steady state in plasma glucose (approximately 8.9 mmol/l) and insulin (approximately 600 pmol/l) was achieved from 45 min to 75 min after meal ingestion (Fig. 2a,b). We can calculate mean glucose clearance in the exercised leg during the 45–75 min quasi steady-state window in the present study as uptake (80.1 µmol kg^−1^ LLM^−1^ min^−1^) divided by the mean plasma glucose concentration (8.9 mmol/l) in each individual to be 9.1 ml kg^−1^ LLM^−1^ min^−1^. This is fairly similar to exercised leg glucose clearance of 10.1 ml kg^−1^ LLM^−1^ min^−1^ in our previous EHC studies when uptake (average glucose uptake 52 µmol kg^−1^ LLM^−1^ min^−1^ [ESM Fig. 1a]) between 45 min and 80 min is divided by the mean plasma glucose concentration (5.1 mmol/l) during the EHC (data extracted from [5]). Thus, compared with our previous EHC studies, the apparently larger leg glucose uptake in the exercised leg in the present study appears to be solely due to hyperglycaemia. The data also indicate that the exercised leg was able to metabolise the incoming glucose at a sufficient rate which is supported by the absence of accumulation of glucose 6-phosphate and free glucose in the muscle biopsies (Fig. 4d,e), which would be expected to occur if metabolism was rate limiting for glucose uptake (see Fig. 6 schematic).

In contrast, in the rested leg, muscle glucose 6-phosphate and free glucose increased after meal ingestion, suggesting that rested muscle was not able to metabolise all the incoming glucose and therefore glucose metabolism was limiting leg glucose uptake (see Fig. 6 schematic). This was reflected in a numerically lower average 45–75 min glucose clearance of 4.3 ml kg^−1^ LLM^−1^ min^−1^ compared with 5.9 ml kg^−1^ LLM^−1^ min^−1^ in the previous EHC studies (data extracted from [5]), where euglycaemia apparently avoided glucose overload of the rested muscle.

Thus, the main reason for the apparently higher exercise-induced muscle insulin sensitivity when measured during a meal test than shown in our previous EHC studies was the much higher glucose uptake that was observed in the exercised leg in this study compared with previous EHCs, while glucose uptake in the rested leg appeared to be similar after meal ingestion vs the previous EHC data, despite the difference in plasma glucose levels between the two tests. This was likely due to the inability of rested muscle to metabolise the amount of glucose transported into the muscle in the hyperglycaemic conditions during the meal test. Indeed, this fits with the higher muscle interstitial glucose concentration in the rested than in the exercised muscle and may also fit with Wasserman and Ayala’s theory that glucose phosphorylation via hexokinase II can be limiting to muscle glucose uptake under some conditions [36]. It is also in accordance with a study showing that the rate limiting step for forearm glucose uptake is metabolism rather than transport when plasma insulin and glucose concentrations are elevated by intravenous infusion [37].

Interestingly, both with meal ingestion and in previous EHC studies, LBF, skeletal muscle TBC1D4^Ser704^ and TBC1D4^Ser588^ phosphorylation, glycogen synthase activity as well as signalling controlling protein synthesis in the previously exercised leg were higher than in the rested leg (Figs 2, 4, 5, ESM Fig. 2 and [18, 34, 35]). Thus, no major differences in muscle molecular signalling were apparent between data from the meal tests and the previous EHC studies. Although not measured in the present study, it is possible that muscle microvascular blood flow was increased more after meal ingestion than during an EHC [18], since GLP-1 was increased after the ingestion of the meals (Fig. 3a) and has been shown to increase microvascular perfusion in human and rodent muscle independently of insulin [38, 39]. If this is additive to the vasodilatory effect of insulin it could contribute to the increased glucose delivery after meal ingestion compared with an EHC. However, given the glucose clearance results, it appears likely that hyperglycaemia following meal ingestion was sufficient to explain the nominally higher leg glucose uptake with the meal test than observed in our previous EHC studies [5], rather than further increased microvascular perfusion stimulated by GLP-1.

Our results emphasise the importance of considering the optimum model to examine questions related to insulin sensitivity. The EHC is considered the gold standard and indeed it is when the goal is to have a constant insulin and glucose concentration to determine glucose uptake at steady state. However, this is not what happens in the ‘real world’ after ingestion of a meal given that blood glucose and insulin concentrations rise and then fall. Indeed, after acute exercise some studies do not find an increase in whole body glucose infusion rate during an EHC [40, 41], but continuous glucose monitoring studies clearly show a reduced excursion of blood glucose with each meal over the next 24 h or so after exercise [42, 43].

A limitation to our study is that the meal test was not compared with an EHC in the same individuals but rather with data obtained from previous EHC studies from our laboratory. We have recently published a meta-analysis of 13 EHC studies completed in our laboratory with the same investigators running the EHCs [5]. Furthermore, the participants in those EHC studies were young, healthy, lean and moderately active men with varying but moderate fitness levels, including fitness levels that characterise the individuals in the present study (Table 1, ESM Table 2). Although the meta-analysis shows rather large inter-individual variation in the response of glucose uptake in both the rested and the exercised leg during the EHC, in our opinion the data in the meta-analysis provide a good estimate of how leg glucose uptake in our meal-test participants would develop during an EHC. For this reason, we did not find it necessary to perform an EHC with participants who completed the meal test for this study. Finally, since the meal-test data and the comparative EHC data [5] were obtained in young, healthy, white men, the results may not necessarily apply to women, individuals of other ethnicities or older adults.

In conclusion, mixed-meal ingestion after exercise results in an apparently greater difference in leg glucose uptake between rested and exercised muscle than we have previously observed in EHC studies. This is likely due to hyperglycaemia following meal ingestion resulting in a large glucose load in addition to robust endogenous hyperinsulinaemia. Evidently, previously exercised muscle is able to dispose of this large glucose load while non-exercised muscle displayed metabolic limitations to glucose disposal (Fig. 6). Our study indicates that when studied with a meal test rather than an EHC, the ability of exercise to increase insulin action on glucose uptake in muscle is bigger than previously thought.

### Supplementary Information

Below is the link to the electronic supplementary material.

Supplementary file1 (PDF 5415 KB)

## Data Availability

Data can be provided upon reasonable request to the corresponding author.

## Abbreviations

A-V: Arterial-venous
DPM: Disintegrations per min
E%: % of total energy intake
EHC: Euglycaemic–hyperinsulinaemic clamp
GIP: Gastric inhibitory polypeptide
GLP-1: Glucagon-like peptide 1
LBF: Leg blood flow
LLM: Lean leg mass
mTORC1: Mammalian target of rapamycin complex 1
p70S6K: p70 ribosomal protein S6 kinase
TBC1D4: TBC1 domain family member 4
TBST: TBS with Tween 20
ULK1: Unc-51-like kinase 1
