# Supplementary material for: Exercise-induced increase in muscle insulin sensitivity in men is amplified when assessed using a meal test
Source: Diabetologia. 2024 Apr 25;67(7):1386–98. doi: 10.1007/s00125-024-06148-x (PMC11153309; doi:10.1007/s00125-024-06148-x)
Supplement: Supplementary file 1 — Supplementary file1 (PDF 5415 KB) [file 125_2024_6148_MOESM1_ESM.pdf]

ESM Table 1 - Overview of antibodies used in western blotting analysis and antibody specifications

	Antibody	Manufacturer	Catalogue no.	Dilution	Diluted in
Total Protein Expression	Akt2	Cell Signaling	3063	1:1000	2% skimmed milk
	TBC1D4	Abcam	189890	1:1000	2% skimmed milk
	p70 S6K	Cell Signaling	9202	1:1000	2% skimmed milk
	ULK1	Sigma-Aldrich	A7481	1:1000	2% skimmed milk
Protein Phosphorylation	Akt^{Thr308}	Cell Signaling	9275	1:1000	2% skimmed milk
	Akt^{Ser473}	Cell Signaling	9271S	1:1000	2% skimmed milk
	$TBC1D4^{Ser588}$	Cell Signaling	8730	1:4000	2% skimmed milk
	$TBC1D4^{Thr642}$	Cell Signaling	8881	1:4000	2% skimmed milk
	$TBC1D4^{Ser704}$	Capra[a]	N/A	1:3000	2% skimmed milk
	$p70\ S6K^{Thr389}$	Cell Signaling	9205	1:1000	3% BSA
	$ULK1^{Ser757}$	Cell Signaling	6888	1:1000	3% BSA
	$mTORC1^{Ser2448}$	Cell Signaling	2971	1:1000	2% skimmed milk

[a]Kindly provided by J. T. Treebak, University of Copenhagen, Copenhagen, Denmark. Remaining antibodies from Abcam, UK, Cell Signaling, USA, and Sigma-Aldrich USA.

ESM Table 2 - Subject characteristics

Age (years)	26	±	2
BMI (kg/m^2)	23	±	2
$VO_{2\ Peak}$ ($ml\ kg^{-1}\ min^{-1}$)	50	±	6

Values are presented as mean±SD (n=106). Data has previously been published [5].

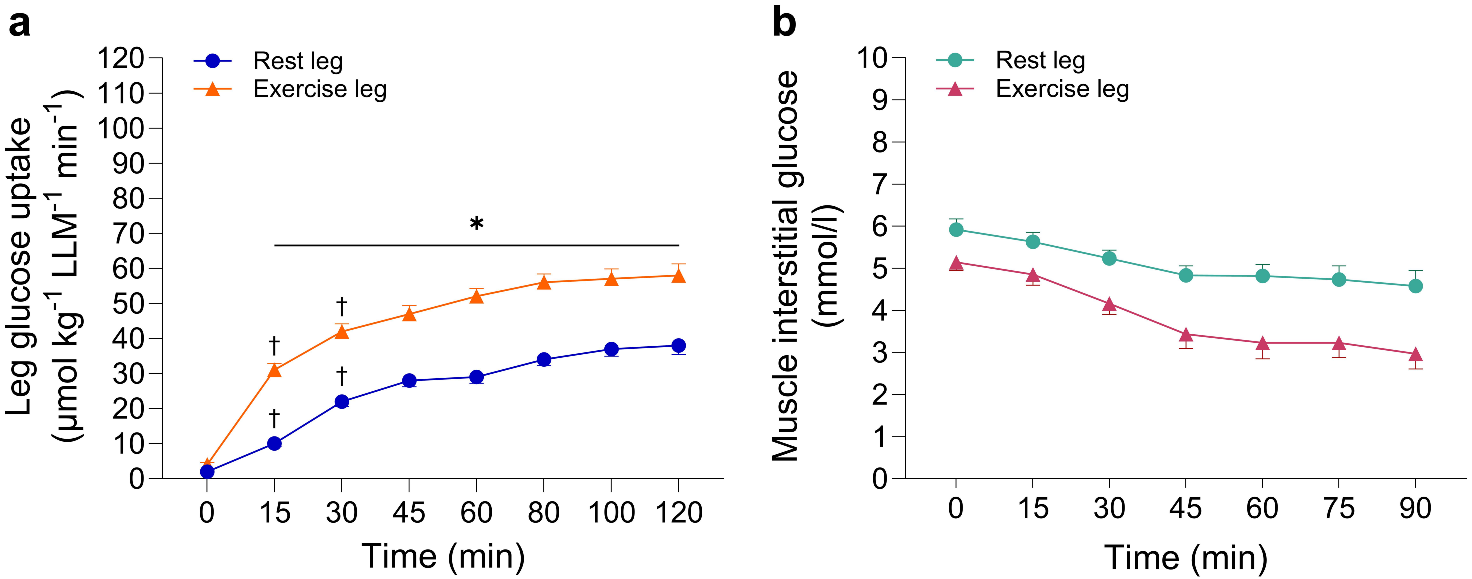

ESM Figure 1. (**a**) Measures of leg glucose uptake during euglycaemic hyperinsulinaemic clamps (0-120 min) reproduced with permission. Data has previously been published [5]. (**b**) Interstitial glucose concentration in skeletal muscle measured during a euglycaemic hyperinsulinaemic clamp (0-90 min) reproduced with permission. Data has previously been published [19]. Data presented as mean ± SEM. Since these data have been subjected to statistical analyses elsewhere, we have not engaged in such analyses here. The statistical notations shown represent the result of analysis performed in the original paper. *p<0.05 for all data points under horizontal line vs corresponding time points for other condition on the same graph; †p<0.05 for data points at the specific time point vs within-condition baseline (0 min).

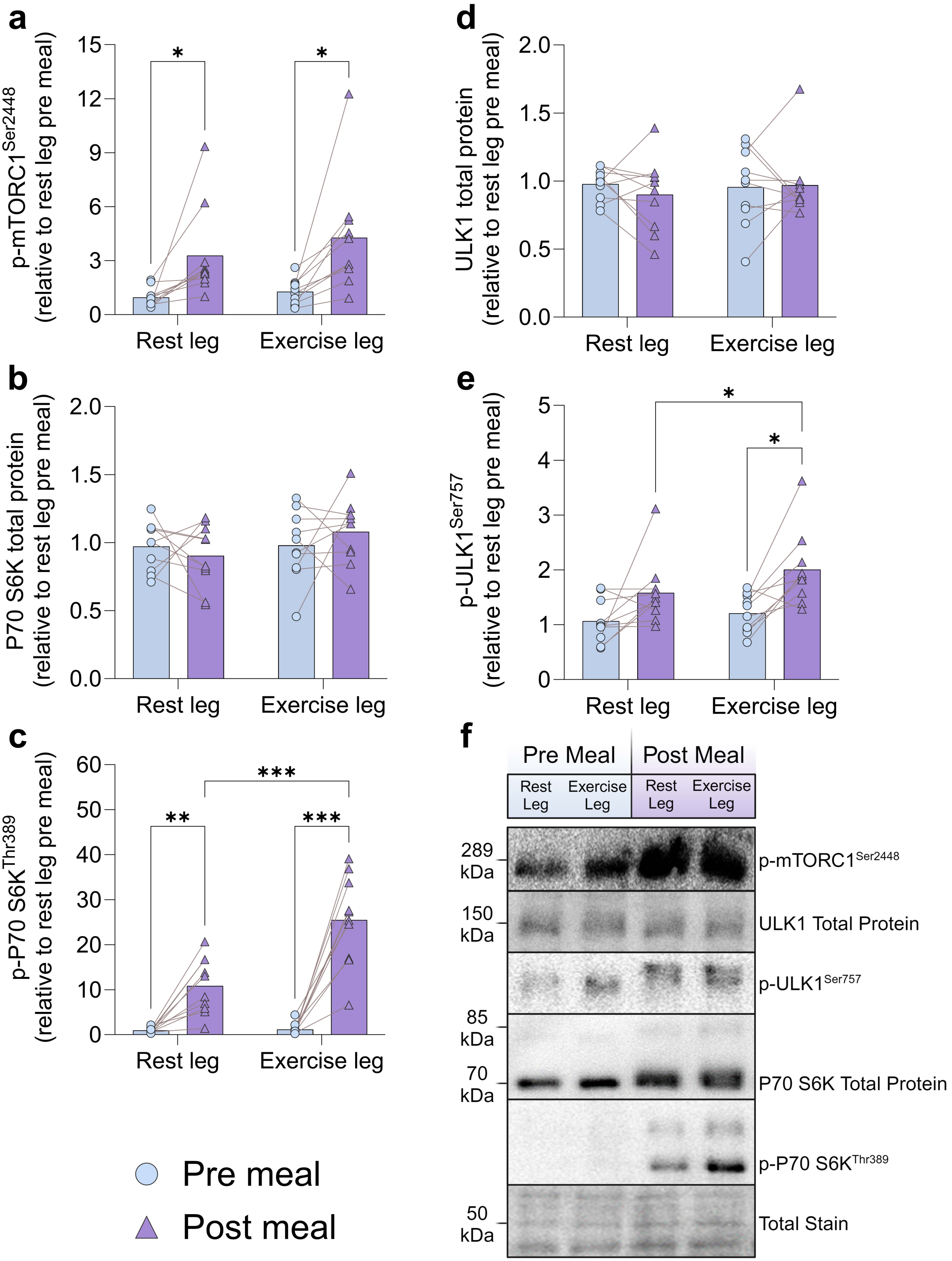

ESM Figure 2. Skeletal muscle molecular signalling in the fasting state and in response to a mixed meal test measured by western blotting. (**a**) p-mTORC1^{Ser2448}. (**b**) p70 S6K total protein. (**c**) p-p70 S6K^{Thr389}. (**d**) ULK1 total protein. (**e**) p-ULK1^{Ser757}. (**f**) Representative western blots for all proteins analysed. Data are presented as means with indication of individual data points. Data is normalised to the mean band intensity of all basal (Pre Meal, Rest Leg) measures on the gel in which it was loaded, n = 10. Statistical significance was evaluated by a two factor analysis of variance and a Holm-Šídák post hoc test. *: $p<0.05$, **: $p<0.01$, ***: $p<0.001$.
